# The Hospital. Nursing Section

**Published:** 1907-01-12

**Authors:** 


### The Hospital.

### Nursing Section.

Contributions for "THE HOSPITAL," should be addressed to the EDITOR, "THE HOSPITAL" NURSING SECTION, 28 & 29 Southampton Street, Strand, London, W.C.

NO. 1,061.—VOL. XLI. SATURDAY, JANUARY 12, 1907.

### Notes on News from the Nursing World.

### A COMPETITION FOR CHRISTMAS, 1907.

THE success of our experiment last year in inviting nurses to write our Christmas Supplement induces us to afford them the opportunity next year of contributing to our columns a special short story bearing upon hospital, infirmary, mental asylum, or district or private nursing life, not necessarily consisting of actual facts, but, if possible, founded upon them. The length of the story, which must, of course, be interesting and probable from first to last, should be about 5,000 words, and, other things being equal, preference will be given to the contribution which is accompanied by two or three good photographs or drawings, for purposes of illustration. The sum offered for the story is £5 5s., and the competition is open to nurses in all parts of the world. In order to stimulate effort, we shall send a cheque for £2 2s. to the author of the story which we consider second best, reserving the right to publish it. With the view of enabling our readers in the Far East and other distant quarters of the globe ample time to forward MS., contributions will be received up to June 30, 1907; but we shall be glad to have them as soon as convenient. They should be addressed to the Editor, Nursing Section of THE HOSPITAL, and marked outside "Christmas Competition."

### TRAINED NURSES FOR THE AMERICAN NAVY.

IT is curious that in America the question of employing trained women nurses for the Navy is still only under consideration. Recently a strong recommendation has been made by the Surgeon-General that Congress be requested to enact a law for the purpose, and it is endorsed by the Navy Department. There is no need to recapitulate the arguments in favour of the proposal, because the naval nursing service in England has long ago justified its existence. We cannot understand why, in this respect, America is behind the times. The value of trained nurses at Haslar and other naval hospitals in this country might be cited as a reason in favour of the establishment of a similar corps on the other side of the Atlantic. It is suggested in the United States that in the event of war female nurses would be needed for hospital ships, but the immediate object in view is their employment for teaching and training the men. ...

### THE NURSING QUESTION IN BURMA.

IT is interesting in connection with the commencement of the operations of the Indian Nursing Association to note that in his report for 1905, just issued at Rangoon, the Inspector-General of Civil Hospitals in Burma dwells on the defective provision of female attendants for the nursing of in-patients, and cordially recognises the claims of the Dufferin Fund and "Lady Curzon's Nursing Scheme" to support. The latter is now merged in the new organisation, and we hope that the Indian Nursing Association will be able, as the Inspector-General expects, to contribute to the settlement of the question of the efficient and careful treatment of females, upon which he believes rests the possibility of securing that medical treatment shall be more frequently resorted to by Burmans than has hitherto been the case.

### OPENING OF A PALATIAL HOME AT CAPETOWN.

THE new Victoria Nurses' Home at the Somerset Hospital, Cape Town, was opened last month by the Governor of the Cape Colony, Sir Walter Hely-Hutchinson. The foundation stone was laid five years previously by the Prince of Wales. It was three years before the Board collected sufficient money to proceed with the building, which has altogether cost £17,000. It contains entrance-hall, dining-room, reception-room, drawing-room, library, and no fewer than 63 bedrooms. The comfort of the nurses has been studied in every detail. The design is in the late French Renaissance style, and the Home is an imposing structure in mountain stone and white plaster, with an internal court. The Governor, in a short speech, warmly congratulated the company on the character of the building, and then presented prizes to members of the nursing staff. The third year prize was awarded to Nurse Lismore; the third year special prize to Nurse Steymann; the second year first prize to Nurse King and the second prize to Nurse Ingram; the first year prize to Nurse Warren.

### BAREFOOT NURSES.

THERE is a small hospital in Roumania, which was started by the European Commission in 1905, where two British trained nurses are working. But for the rest the nursing in that country is very much behind the times. The Queen, Carmen Sylva, is greatly interested in the poor, and does her best to raise the standard. But the persons engaged in nursing belong chiefly to the lowest class of servants. In a general hospital, considered the best in one of the principal Roumanian towns, the nurses, if such they can be called, stalk about in the corridors most untidily dressed in loose blouses and bare feet. A great many of the patients are strapped down, and the others appear to attend to themselves. Owing, perhaps, to ground in Roumania being cheap, there is always a large garden attached to a hospital, and the exterior of some of the buildings is imposing, with wards constructed in the English style. We are afraid that until a system of training is introduced there will be little improvement in the existing deplorable nursing arrangements.

### THE RESIGNATION OF MISS ELKINGTON.

It is a little more than ten years since Miss Charlotte Elkington became the Lady Superintendent of the Queen's Hospital at Birmingham. Her retirement, which we announced last week, takes effect at an early date. Miss Elkington was trained at St. Thomas's Hospital, and at the end of her training worked for three years under the auspices of the National and Metropolitan District Nursing Association. In 1888 she returned to St. Thomas's Hospital as sister of the Victoria Ward, and in 1894 she was promoted to the position of assistant-matron. In 1896 Miss Elkington went to Birmingham, and during her tenure of office as Lady Superintendent of the Queen's Hospital she enjoyed the confidence and affection of the whole of the nursing staff, as well as the respect of all with whom she has been brought into contact.

### GLASGOW ROYAL INFIRMARY.

At the New Year's Day meeting of the directors of Glasgow Royal Infirmary, the Lord Provost having referred to the recent death of Nurse Bella, a devoted worker in the institution, expressed his regret that Mrs. Strong, the matron, who has done so much for the status of the trained nurses, has been compelled to take a rest from her labours for a time. He was sure that they all trusted she might be soon restored to health. Mr. J. D. Hedderwick, chairman of the directors, while joining in the Lord Provost's expression of regret at the enforced retirement of Mrs. Strong, said he was glad to know that a most efficient substitute had been found in Miss Melrose. The Chairman also made the welcome announcement that the architect has been instructed to proceed with the Nurses' Home, and that the Board hoped shortly to reinforce the nursing staff, which is admittedly under the proper strength.

### THE INSPECTOR OF MIDWIVES FOR SOMERSETSHIRE.

With regard to the appointment of an inspector of midwives by the Somersetshire County Council, to which we recently alluded, the new official, Miss E. C. du Sautoy, who entered upon her duties last Friday, is to give her whole time to the duties of her office, "except that she is at liberty to place such portion, as may be agreed upon from time to time, at the disposal of the Somersetshire County Nursing Association, who undertake to make the same good to the County Council by providing, free of expense, to the Council—save travelling expenses—during such period a competent deputy, to be approved by the Council." It will thus be seen that, although she may not officially hold the position of superintendent of the Somersetshire County Nursing Association, Miss du Sautoy will practically act in that capacity. It remains to be seen how far the dual appointment will work out satisfactorily to the Somersetshire County Council, the County Nursing Association, and Miss du Sautoy herself.

### INDIVIDUAL GUARDIANS AND NURSES' GRIEVANCES.

In a letter which appears on another page a well-informed correspondent mentions among the drawbacks of the existing system of workhouse nursing, the airing of petty grievances by junior nurses of a certain type who find that they can interview individual guardians. This drawback is illustrated in the state of affairs prevailing just now at Newcastle-on-Tyne, where friction and resignations are rife at the Workhouse Hospital. One of the chief causes, it is stated, is the disposition of individual guardians to listen to the story of a nurse's grievance. The nurse resigns, tells a guardian the reason, he raises the case at the next meeting, a discussion ensues, and the nurse is asked to withdraw her resignation. She complies, and the institution rings with the tale of her moral victory, which fires others to emulate her example; and thus indiscipline is promulgated. The inevitable result is the increasing absence of discipline in the wards, and frequent changes in the nursing staff. Nurses should be absolutely prohibited from making complaints to individual guardians, and the superintendent should be supported by the Board as a mere matter of ordinary routine.

### A WORKING MAN ON A NURSING ASSOCIATION.

We are glad to see in the *Darwen News* a letter by a working man calling attention to the claims of the Darwen Nursing Association. He dwells on the good work which he knows that the nurses are doing, and then contrasts the handsome sum of £460 collected from the Darwen mills and workshops during the year for the Blackburn and East Lancashire Infirmary with the meagre amount of £30 obtained for the Darwen Nursing Association. He does not, of course, think the former too much, but he considers that the latter is not at all in due proportion to the good which is done in Darwen by the nurses. It is not creditable to the town, he adds, that not more than one-half of the mills and workshops contributed anything during the twelve months, and he appeals to the masters of the mills to take up the matter and show a personal interest in the work. Such an appeal can scarcely fail to have a good effect, and to render the sum contributed by the working people of Darwen to the support of the Nursing Association more worthy of them. There are other towns where a working man might personally plead for help to a similar cause.

### AFFILIATION OF THE STAFFORDSHIRE ASSOCIATION TO THE JUBILEE INSTITUTE.

It was announced at the last meeting of the Staffordshire County Nursing Association that the Executive Committee had applied for affiliation to the Jubilee Institute. The organisation was only formed last summer, and the total amount received or promised so far is £121 15s. in subscriptions, and £24 11s. in donations. Lord Harrowby is chairman of a representative committee, who are about to appoint a qualified county superintendent. We have no doubt that the affiliation of the Association to the Jubilee Institute will not only be for the benefit of the patients nursed under the auspices of the Association, but will also tend to quicken the flow of contributions to its support.

### A PRECAUTION FOR MATRONS.

A Report has been issued by Mrs. Grace Neill, officer in charge of St. Helens Hospital, who has been investigating complaints concerning the management of St. Helens Maternity Hospital at Auckland, New Zealand. The complaints were made chiefly by two ladies, who averred that the matron had refused admission to patients who considered themselves eligible, and that there was friction in the institution between the matron and her staff. Mrs. Neill affirms, in reference to the former accusation, that there was no ground for it; but in order to avoid misunderstanding in the future she has instructed the matron to record in a book the name and address of every inquirer before answering any questions. With respect to the latter the only friction she discovered, after being in and about the hospital at all hours for ten days, was due to the grumbling of a single pupil nurse, who was out of harmony with her surroundings, and she suggested as a remedy that the dissatisfied young woman, who voluntarily entered the hospital, should voluntarily take her departure from it.

### PRIZE DISTRIBUTION AT TAUNTON HOSPITAL.

Many compliments were paid by speakers at the distribution of prizes to the probationers of Taunton and Somerset Hospital to Miss Bulteel, the matron. The Hon. Mrs. Stanley, whose name is a household word among the nurses of Somersetshire, expressed the pleasure which it afforded her to perform the ceremony of giving away the prizes and certificates, and concluded by saying, "God bless you in your work and prosper you." The prize for elementary anatomy was awarded to Nurse Smith, and certificates of merit to Nurses Olver, Littlejohn, and Nickson; the prize for nursing to Nurse C. Fox, and certificates of merit to Nurses Melton and Nickson; while the prize for elementary physiology was won by Nurse Hines.

### HOSPITAL BAZAAR ON THE PAVEMENT.

It may be remembered that three years ago Townsville, North Queensland, was visited by a terrible cyclone, and the most important part of the hospital was demolished. At the same time some of the bedridden inmates perished and two nurses were injured in getting their patients out from the tottering building. The new hospital, which has been erected in the place of the one which was destroyed, is a very fine two-storied building, and includes, as well as the usual wards, three private ones where paying patients are received at £3 3s. a week, and others which are specially set apart for Chinese and Japanese sick. As the hospital was imperatively in need of money a novel kind of bazaar was organised in November, and was open from 9 a.m. to 11 p.m. The stalls, the contents of which included plain and fancy work, paintings, wood-carving, flowers, fruit, dairy produce, eggs, cake, live-stock, including ducks, fowls, and sucking pigs, were set out along the side of the pavement, and business was so brisk that nearly £400 was realised. Raffles for £5 notes and for jewellery were popular. As the Government gives 30s. for every 20s. collected privately, the sum at the disposal of the Townsville Hospital will be nearly £1,000.

### NIGHT DUTY WITHOUT REFRESHMENTS.

The Shepton Mallet Guardians have decided not to act upon the suggestions made by Mr. Preston Thomas, the Local Government Board Inspector, that they should appoint two assistant-nurses to take alternate day and night duty in the place of one assistant-nurse and a night nurse, the latter coming in from outside. It is not disguised that the reason for their decision is chiefly that if a second permanent nurse were appointed the Guardians would be obliged, in accordance with the orders of the Local Government Board, to have a superintendent nurse. They are therefore continuing the old arrangement, though they have agreed to move one step forward. In future the nurse from outside, when she is on night duty, is to be allowed part rations; previously she has not been supplied with any refreshment.

### THE DRAMA AND DISTRICT NURSING.

The handsome sum of £265 was realised by five performances of "The Physician," recently given by Hudson's Dramatic Company at West Bromwich, in aid of the District Nursing Association, whose headquarters are the Akrill Home. Mr. Hudson himself generously discharged the whole of the unavoidable expenses of the performances, so that the entire proceeds have been handed over to the Treasurer of the Association. But for this great addition to its funds, the year 1906 would have ended with a debt of £7. While we congratulate the Association on the help it has thus received, we hope that there will be no slackening, in consequence, in the essential work of augmenting the list of regular subscriptions.

### ENLARGEMENT OF A NURSES' CLUB.

The Residential Club for Nurses at 129 St. George's Road, Pimlico, which was opened about two years ago, has been enlarged. The house at the back has been secured and connected by a covered passage with the original building. The annexe consists exclusively of private rooms, all of which are let to members. Particulars of the very moderate tariff were given in our issue at the time the club was started, and the extension justifies the belief that it supplies a want. At the outset there was a manageress, and the Club was run by a committee until July 1906; but the two ladies to whom it belongs, Miss H. Mengens and Miss Tabor, are now exclusively responsible for the management, Miss Mengens acting as superintendent. Only nurses are received as members of the club, but the latter are permitted to introduce friends for a visit or for a meal at any time.

### SHORT ITEMS.

A Concert given last month in aid of the Napier-Clavering Memorial Nurses' Home at Blaydon-on-Tyne, yielded a sum of £15, which has been handed over to Nurse Wilson.

### The Nursing Outlook.

"From magnanimity, all fears above;
From nobler recompense, above applause,
Which owes to man's short outlook all its charm."

### NURSES' HOURS AND NURSE TRAINING.

A FORTNIGHT ago we dealt with the question of nurse training and hospital expenditure. The facts that the cost of nursing in hospitals is steadily increasing, that the number of nurses and probationers employed each year in many hospitals with nurse training schools is multiplying, and that the question has been raised whether it is legally justifiable to devote hospital funds to the training of nurses other than those required for the purposes of each hospital's necessary work, were dealt with. It was further held that a remedy might be found by charging a reasonable fee to extra probationers for their training, whereby all cost to the hospitals would be avoided, whilst the admission of these additional probationers might be attended by many advantages. One of these advantages, the reduction in the number of hours each nurse is on duty, we propose to now consider.

Hours of duty have undergone many changes, and the quality and character of the work entrusted to nurses in hospitals has also materially changed. Twenty years ago there were many too few nurses and the hours of duty and food supplies at many hospitals left much to desire. In 1890 in some of the best and largest hospitals in the country nurses were on duty from 7 A.M. to 9 P.M., with the exception of an hour and a half allowed for their meals. At St. Bartholomew's Hospital, for instance, on three days, and sometimes on four days in each week, the nurses were kept on duty for fourteen hours in each day with no intermission except at meal times. Yet Miss Isla Stewart, the matron, held that fourteen hours of such work did "not seem such a hardship." For she wrote "there is apparently no help for it at present, as "I doubt if any of the authorities in hospitals would "feel justified in incurring the enormous expense "which would necessarily be entailed by the in- "crease of the nursing staff sufficiently to allow of "shorter hours."

To-day the position of nurses in respect to hours of duty in many hospitals has greatly improved. This result is due to an increase in the leave allowed, as at Guy's, the off-duty time granted to probationers averages at least two hours every day with two half days and one whole day in four weeks. The head nurses or sisters have in addition one Sunday, to precede or follow the whole day off duty. The whole day and half days off duty are arranged to fall on the days on which, in the ordinary course, the nurse would have three hours off duty. Put tersely the hours are so arranged that, on an average, each nurse and probationer is on duty for between eight and nine hours. Having regard to the fact that, in a well regulated hospital, during the hours of duty, unless the work of a ward is for the moment exceptionally heavy, considerable pauses may and in fact do occur, when the nurse on duty has some leisure at any rate, less than nine hours on duty is an average minimum which may be accepted as satisfactory from the nurse's point of view at any rate.

We have been speaking so far of the larger hospitals in London. The case of the provincial and Scottish hospitals is, with few exceptions, slightly different. In those hospitals where there are no medical schools the work is usually much lighter than in others. In clinical hospitals the students do much of the work which devolves upon the nurses at the smaller institutions. In the latter the work which a nurse has to undertake is often much more practical and sustained. We find that nurses' hours on duty outside London, with few exceptions, are greater than in London itself at the present time. Ten to eleven hours on duty would seem to be about the average, and at first sight it may be thought that these hours compare unfavourably with those at the Metropolitan and a few of the larger provincial and Scottish hospitals. The work of the provincial hospitals is, however, less exacting from the nurses' point of view, owing to the fact that the honorary medical staff visit the wards in the early part of the day, so that in the afternoon the work is light and the nurse may often have much more time to herself. In a large London hospital the honorary medical staff usually visit the wards in the afternoon, a practice which greatly increases the strain on the nurses employed.

We may take it, therefore, that the hours on duty of nurses generally throughout the country are not now excessive. How far the reduction, which has taken place in these hours in hospitals with nurse-training schools, may be due to the admission of extra practitioners, there is no evidence to show. The point is one however well worth considering by the economy committee of each hospital. Another interesting point would be to ascertain, how many extra probationers would be required, in each hospital, to render it possible to everywhere limit the hours on duty for every nurse to eight per diem? We imagine, in view of the facts here stated, that very few extra probationers would be required in the majority of hospitals, and that if they were employed then the maximum expenditure on the nursing staff would have been reached. If so, each economy committee would then be in a position to closely consider the question of cost, with the view to its reduction to reasonable and fixed proportions, by certain modifications introduced into the existing system of training probationer nurses.

### Nursing in Tropical Climates.

By ANDREW DUNCAN, M.D., B.S., M.R.C.P., F.R.C.S., Fellow King's College, Lecturer on Tropical Medicine at the London School of Tropical Medicine, and the Westminster Hospital.

### IV. ENTERIC FEVER.

WE now come to the subject of enteric fever in the tropics—perhaps the most important one in the whole range of medicine as regards nursing; for in this disease a good nurse is invaluable, and in no disease is efficient nursing more demanded. In the absence of any of the sudden complications requiring skilled medical aid, an able nurse is far more important than the medical attendant.

This affection is one of the most frequent and most serious to be met with in India, and in consequence of its excessive prevalence, from time to time various curious theories have been held to explain its frequency. These, however, need not detain us. At one time, strange to say, it was considered by the authorities not to exist in India at all. In 1853 it was discovered by Scriven at Lahore in Europeans; in 1856 it was found amongst the natives by Ewart in the Ajmere Jail; and in 1859 it first obtained a place in the statistical returns. There has, until of recent years, ever been the opinion that natives were scarcely affected; but this has, since the employment of the Widal test, been shown to be erroneous. No race or creed is exempt. The merit of demonstrating its frequent occurrence in natives is due to chiefly Wright of the 6th B.I., Elliott of Madras, Buchanan of Nagpore, and Leonard Rogers of Calcutta; the latter indeed stating that over 80 per cent. of the cases of continued fever, lasting as long as three weeks, proved to be enteric fever on using the Widal test. The application of this test has in truth revolutionised our ideas of the occurrence of this fever amongst natives. There is much controversy in India as to the method by which this affection originates; the consideration of this, however, need not take up our time; suffice it for the nurse to know that enteric fever may be communicated by the ingesta, by the excreta, by flies, and by dust. To emphasise these points, a few examples may be quoted. One need not mention cases caused by the ingesta, as these are well known; nor need one emphasise the part played by the solid excreta, beyond stating that by this time the soil of India must be fairly impregnated with the bacillus of Eberth. And be it recollected that the bacillus is possessed of great vitality. Cayley records a case where the typhoid stools were burnt in a dunghill. Some five weeks after five persons were employed in removing dung from the heap, and were attacked with enteric. Their alvine discharges were buried deeply in the same heap. Nine months subsequently two men were employed in the removal of the heap, one of whom was attacked with enteric fever and died. But besides the solid fæces, the urine is a fertile source of conveyance, as shown by Dr. Horton Smith in his Gulstonian lectures. And here the nurse should remember that the specific bacilli persist in the urine long after the defervescence of the fever. For instance, Dr. Lazarus has found them 41 days in the urine after the temperature had attained the normal. Dr. Petruscky gives a very illustrative case, showing how the urine can be infectious in man. The sister of one of his wards accidentally drank a small quantity of urine which had been passed by a typhoid fever patient into a wine glass, and after an incubation period of 14 days she developed typhoid fever. Urine is probably one of the most frequent causes by which the soil of India is contaminated.

A fertile source of infection is through the dust. Anyone who has experienced a dust-storm in India knows how difficult it is to keep it out of one's bungalow, and should the dust be infected, one can understand how this, being blown on to the food therein, will infect it in its turn. At Quetta in 1898 an epidemic occurred. No case was admitted until May, when the usual dust-storms began. The outbreak was preceded by sore throat from the dust, and several cases began with sore throat. The filth-pits were to windward of the barracks, and the epidemic was worst in the lines nearest the pits. Again, in an epidemic at Lundi-Kotal, in the Khyber Pass, the affection began in June, when the dust-storms commenced to rage. At the end of July, when the excreta were removed for disposal one and a half miles from camp, the epidemic began to cease. As regards the agency of flies, their importance was well shown in the Spanish-American war of 1898. A Commission was appointed to examine into the causes of the great epidemic that had occurred, and it stated that "flies were undoubtedly the most active agents in the spread of typhoid fever. Flies alternately visited and fed on the infected fæcal matter and the food in the mess tent; typhoid fever was much less frequent among members of messes who had their mess tents screened than it was amongst those who took no precaution." Again, in our South African war flies swarmed everywhere, and seemed to be particularly attracted by the enteric fever tents, and our medical staff held they were active agents in the dissemination of the disease. It is needless to state what an ever-present plague they are in India, especially in the hot weather. Before concluding the subject of the methods of transmission, it may be mentioned that much evidence has been given of late years, especially by Dr. Collie, of the Homerton Fever Hospital, that enteric fever can be contracted by direct contagion. With these preliminary remarks let us now pass on to the actual nursing of an enteric fever patient.

*The Nursing of an Enteric Fever Patient.*—During the evolution of enteric fever the chief stress falls on the structures known as Peyer's patches in the small intestine; these become inflamed and swollen, and slough; when the slough is cast off ulceration is left behind, which, as the case goes on to recovery, heals up. But on the other hand the sloughing process may not cease, but extend deeply through all the coats of the intestine, during which hæmorrhage may occur from a small artery being laid open; as the extension goes on, perforation finally occurs into the peritoneal cavity. Hence the nurse should always bear this danger in mind; and hence the necessity of the careful regulation of the diet, and the avoidance of all unnecessary movements of the patient.

*Management of the Sick-room.*—This must be kept as cool as possible; in the hot weather you cannot keep your windows and doors open, as the room will become unbearably hot: punkahs, and thermantidotes where available, will, of course, be in use. The patient should wear a thin night-shirt, best made to open down the back, and fastened by tapes. The bed-clothes should be light, consisting of a sheet only over the patient; a blanket is not necessary, as there is no fear of catching cold. In the Punjab and North-West, should the disease be contracted in the cold weather, during convalescence the bed-clothing must be increased. The cummerbund should be worn. The pulse and respiration rates will, of course, be taken, and the temperature range noted; the latter should be taken every four hours. And here it may be remarked that the nurse will not in my experience see the range exhibited after the manner of cases occurring in a temperate climate. This range is, in the cases I have seen, very exceptional. The temperature in India follows no very settled course, but on the whole may be described as irregularly remittent.

*The Question of Feeding.*—This is of the greatest importance. The attending physician will order the quantity; the nurse should be careful never to give the patient a large amount at one time. Milk, if it agrees, will form the diet, and here the nurse must carefully watch for the appearance of any curds in the stools, for if present they indicate that it is not being properly digested, and the physician must be informed of this at once. With regard to the food, Dr. Hale White has pointed out that the usual amount of milk ordered—namely, 3 pints in the 24 hours—does not supply the necessary amount of calorics of energy per diem. (A caloric is the amount of heat necessary to raise one kilogramme, or 2.2046 pounds, of water from zero temperature C° to 1.C°.) Three pints of milk only supply 1,350 calorics of energy; he therefore recommends that in addition a tablespoonful of maltine or honey should be given four times a day, for this quantity of maltine supplies 120 calorics; hence, by giving four tablespoonfuls you give 480 calorics, or, with the milk, 1,830 calorics; this is practically sufficient, for a patient should have enough food to give him 2,000 calorics of energy per diem.

Should masses of curds be found in the stool, the milk should be given peptonised, or the milk may be converted into whey, by boiling each pint of milk with one or two tablespoonfuls of lemon juice, and straining through muslin, strongly squeezing the curd. In this process, however, we lose the albuminous constituent in the curd, but to make up for this you beat up a new laid egg and add it to four ounces of the whey, about three times in the day.

Dr. Burney Yeo has drawn attention to the fact that in very many cases milk will not agree with enteric fever patients, as shown by their vomiting a firm cheesy mass soon after taking the milk, or by the continual passage of motions with masses of curd in them. Under these circumstances the milk should be tried after diluting it with twice its quantity of water; if it still disagrees, freshly made clear soup may be administered, or dilute albumen water made with white of eggs (the whites of two eggs with one pint of water, foamed up and strained). He narrates one very severe case complicated with alarming hæmorrhage, in which the patient was fed on one teaspoonful of Valentin's meat juice in a wine glassful of cold water every three hours with one teaspoonful of brandy. This was the only diet given for seven days, and answered admirably. These points are, however, only mentioned to complete the subject of feeding, as, of course, the doctor himself will give all the necessary orders for the method and lines of feeding. Finally, with regard to stimulants, in my experience they are more often called for in the Tropics than in temperate climates.

*Clinical Course of Tropical Enteric Fever.*—The nurse must not expect to see this fever run a course similar to cases in temperate climates. This fever should always be held in mind when anybody has had a temperature uninfluenced by quinine for a week. The temperature, as before remarked, does not show the usual type of entĕric fever at home as a rule. Other differences in the course of the fever are the following: *Diarrhœa* is less marked, and often occurs only at late periods in the third or fourth week; the *rash* is stated to occur less frequently; *the characteristic tongue* is not so often seen; *gurgling* and *abdominal tenderness* are less frequently observed; *cardiac failure* and *severe pulmonary complications* more often occur; *thrombosis of the large veins* is said to be met with more often; the *onset* is perhaps more rapid.

### The Nurses' Clinic.

### DRESSING OF SIMPLE WOUNDS.

Both in hospital and private practice, and certainly in district nursing, it usually happens that minor surgical dressings are left for the nurse to do, the doctors reserving to themselves the more important aseptic dressings which involve serious responsibility and wide experience. Though nurses meet with a great variety of wounds in the course of their daily work there are certain practical points which are essential to the successful dressing of every kind of wound, and without which healing will be delayed, if not indefinitely protracted. First, then, whether it be a case of burns, chilblains, ulcers, cuts, abrasions, or otherwise, let everything that is necessary for the dressing be in readiness before the bandage is removed; this will save time in the end and save the patient's temper and feelings as well, not to mention the fact that no wound is the better for being exposed to the air longer than is absolutely necessary. With regard to the kind of dressings to use, if a wound is discharging it is always safe, in the absence of special instructions, to apply a hot boracic fomentation and continue it until the wound is "clean" and shows the faint blue line of healing, which is so easily recognised by nurses. Then we may try boracic ointment or lanoline and zinc ointment. The latter ointment has a wonderful property of drying up sores, but it is too irritating for some

THE NURSES' CLINIC.—*Continued.*

varieties of skin. Whenever ointment dressing is used let the piece of lint be no larger than the circumference of the wound so that the surrounding skin may not be unnecessarily irritated. Sometimes a wound which has commenced to heal will do better with a dry dressing, then we may try boracic lint, and leave off daily applications. It is not always of vital consequence what kind of application is used in the healing of wounds, though judgment and experience are the best guides in the matter; the main points are to keep the wound clean, exclude the air from it, and supply the necessary rest and support during the process of healing.

When a scab forms it must be removed if there is any reason to suppose that pus may have formed underneath, otherwise the scab may be left until it wears off, as it will probably do when the wound is healed. Now and again we have to dress sloughing ulcers such as develop from neglected bed-sores; these are often very offensive and will need some deodorant to cleanse them. The doctor will perhaps order charcoal and linseed poultices; these are made like ordinary linseed poultices with charcoal sprinkled over them. Sometimes a daily bath is useful for the removal of offensive discharge; in any case local immersion is soothing and comforting to the patient. Should a wound have been caused by a dirty instrument or rusty nail, or by a fall upon the ground, great care must be taken to cleanse it thoroughly before the dressing is applied, as no wound will ever heal while any dirt or irritating matter remains in it. In the event of gaping a piece of strapping with incisions—like a gridiron—made in it will draw the edges together and assist healing, the part with the incisions of course being immediately over the wound to allow of discharge escaping. Should there be serious gaping the nurse will summon a doctor, who will decide whether stitches are necessary, and, if so, insert them. In the dressing of blisters the skin should not be removed unless the doctor has specially ordered the blister to be kept "open," the nurse should lightly snip the skin in the most dependent part to allow the fluid to escape, and then dress with some simple ointment such as zinc or boracic. A word must be said on the subject of burns. Sometimes it is more important to put the patient, especially if it be a child, to bed, and give him a hot drink than to dress his burns at once. Even apparently slight burns cause a shock to the system, and the shock needs to be treated before the burns. I have known an infant's death caused by the falling of a cinder or two into its cradle, the cradle having been unwisely placed too near the fire; the burns were very slight, indeed, but the baby died notwithstanding. Some burns appear quite simple at first sight, but often develop into serious wounds a few hours afterwards, so that we need to be on our guard not to decide too hastily, lest complications ensue. Whatever dressing is applied to a wound, the bandage should be fixed firmly enough to keep the dressing in its place without being tight enough to cause pain to the patient. A bandage which slips out of place is the mark of an incompetent nurse, and is the cause of discomfort to the patient, even if no worse result follows. I read only recently of a case in which an abdominal wound was entirely exposed the day after an operation; to whose carelessness this fact was due history does not relate.

In all contact with wounds the nurse must be very careful not to infect her own fingers. The tiniest cut or scratch should be rigorously painted with collodion or otherwise covered with an india-rubber finger stall to prevent the possible entrance of poisonous germs, and the hands should be thoroughly washed and disinfected both before and after dressing a wound. A nurse should never, however hurried, touch soiled swabs with her fingers; the writer once knew a nurse who lost the use of three fingers of her right hand by neglecting to use her forceps for gathering up soiled dressings. Instruments need as careful washing and sterilising as hands do, especially forceps with corrugated edges, in which microbes may lodge so easily. Every vessel that has been used for a dressing should be conscientiously washed and disinfected, so that no infection may be carried to the next case. And after we have done our part let us console ourselves that nature is stronger than we are, and will do more than we can ever do towards the work of healing. We only need to place the patient in the best possible condition for recovery, and nature will do the rest.

There are, however, some cases so obstinate that they get the name of "chronic ulcers," and are the despair of doctors and nurses alike. If they do manage to heal they break down again upon the slightest provocation, and we have to go over all the ground again. In that case, perhaps, the best thing is to place the patient in the fresh air and try open-air treatment and good nourishing food, and perhaps with improved nutrition even "chronic ulcers" would heal.

### Incidents in a Nurse's Life.

### THE NEW YEAR'S GIFT.

It was New Year's Eve. Outside the fog hung thick and heavy over a densely-populated district in the East End of London. Within the women's ward of a hospital, situated in one of the poorest localities, all was warm and comfortable. The fires in the open grates at each end of the ward gave out a cheerful glow, casting fitful shadows on walls and beds, and throwing into relief the various groups of multicoloured flags adorning the walls, which were almost all that remained of the Christmas decorations, the holly and mistletoe having been taken down some days previously for reasons "antiseptic." Sister had read prayers, and the nurses had lowered the lights. The clock of a church in the neighbourhood chimed the half-hour after eight. Soon the night nurse would make her appearance, and, in the meantime there was the quietest time in all the busy day to be enjoyed—the short half-hour which succeeds the lowering of the lights, and precedes the coming on duty of the night staff. Sister sat down at her writing-table. By and by she and her nurses, along with many others of the staff, would troop out to the neighbouring church for the Watch-night Service.

Presently a footfall was heard at the ward door, and a house-surgeon made his appearance.

"A case coming up for you, sister; erysipelas of leg and"—here he spoke in a lower tone, and sister looked up sharply—"but," he continued, "it is not expected to come off for two months, and we shall have her leg well, and pack her off home long before that time."

"But," expostulated sister, "it is down in the rules in black and white that these cases are not to be admitted."

"Can't help it, sister; we can't turn the woman out with a leg in that condition. Besides, she has nine children at home, she tells me, and a husband out of work. So I'll send her up. Let her have boracic fomentations to the leg every four hours."

With a cheerful grimace, and humming a tune, the genial house-surgeon took his departure.

A bed was hastily covered with bathing blankets, and the patient was soon in it. Sister took her temperature, and the night nurse, who had arrived in the meantime, proceeded to blanket-bath her. This being accomplished, the bad leg, which was hot and inflamed from toes to hip, was enveloped in a soothing fomentation, and the patient was soon warm and snug, though suffering considerable pain. Night sister made her appearance and received the report, at one part of which she also looked up as sharply as sister had previously done when the house-surgeon came up. "I hope it won't come off this side of two months for the woman's sake," she whispered; "it will be a nice state of affairs if it does, with a leg in that condition."

Eleven o'clock came, and the church-going party left the hospital in twos and threes. The service ended, they trooped back through the fog, congratulating each other that they had not far to go on such a miserable night, and looking forward to the hot coffee which had been promised them on their return by the night nurses.

The genial house-surgeon was waiting in the hall when sister arrived.

"A Happy New Year, sister. I've got a New Year's gift for you upstairs. Come along and I'll show it to you in your ward."

With a mischievous smile he led the way, followed by the perplexed sister.

Straight up to the new patient's bed he went, and sister was surprised to see it closely surrounded by screens. "Come along inside," he said, now thoroughly enjoying her perplexity.

There was the new patient, and by her side nestled a little bundle of pink humanity, clothed in lint and wool, and wrapped in a blanket in lieu of the orthodox baby-clothes.

"What do you think of that for a New Year's gift, sister?" he said.

"And you told me it was not expected for two months," rejoined sister, in a grieved tone.

"I told you exactly what the patient told me, but you had better have it out with Mrs. 16 to-morrow. From the complacent look on her face I suspect she knows a thing or two, and I only hope," he added in an undertone as he turned to go, "she won't get any complications."

Certainly Mrs. 16 looked as if she did know a thing or two, judging by the smile of intense satisfaction which irradiated her countenance. Maybe she contrasted her surroundings on this event, with nine similar occasions. At any rate, she evidently did not suffer from the fear of dreaded complications which exercised the minds of those in whose charge she had so unexpectedly landed. Puerperal fever loomed not on her mind's horizon.

The next day was a busy one for sister. A cot was improvised out of two wicker chairs. Lint, wool, and bandages did duty for baby-clothes until there was time for these to be procured. Feeding-bottles were requisitioned, for "The Gift"—as the baby was dubbed—was not allowed to be fed by his mother for obvious reasons.

The unexpected little stranger throve amain, and was the pet of the ward. Great amusement was caused amongst the staff when sister announced that he was going to be christened in the ward, and that she had asked the house-surgeon to be god-father. The house-surgeon did not relish the honour, protesting that, being a Scotsman, he himself had neither god-father nor god-mother, and that he really could not officiate in that capacity. In vain sister pointed out that it was his duty, being in charge of the case. He stoutly refused the honour, and "The Gift" was deprived of his guardianship.

Two Christian names were bestowed upon the babe, one being the name of the ward in which he was born. The mother got on splendidly without any complications, and the leg was sound again in a month.

### New Year and Christmas Entertainments.

### An "At Home" at Charing Cross Hospital.

The festivities at Charing Cross Hospital concluded with an "At Home" given by the residents and nursing staff on January 2. The large out-patients' hall was well filled with a thoroughly appreciative audience, who listened with enjoyment to an excellent concert, arranged by Dr. Fenton. The programme contained the names of several professional artists as well as a number of volunteers. Such patients as were sufficiently convalescent to attend were accommodated with seats in front, and appeared to enjoy the whole performance exceedingly. There was no lack of humourous recitations and sketches in the course of the evening. Mr. Morris Harvey convulsed his audience by his clever imitations of all the great lights on the stage, and Mr. Margetson's account of the wanderings of the popular air "Bill Bailey" until its final adoption in Germany by a certain august personage as the "leit motif" of his new opera, was received with shouts and merriment. Mr. Lewis Sydney, late of "The Follies," also very kindly contributed his quota in the shape of a string of funny stories, each one funnier than the last, and a laughable sketch of a distressing scene in a ball-room. The Rev. Harold Davidson is always a welcome performer on these occasions, and his two recitals were hailed with much applause. The musical part of the programme was of an equally high order, Mr. Hubbard's songs being well received, while Miss Helen Mott on the violoncello and Mr. Edgar Wilby on the violin gave great delight to the audience.

### St. Bartholomew's Hospital.

The Christmas dinner at St. Bartholomew's Hospital was a grand affair—turkeys and geese and plum-puddings galore. At four o'clock the excitement reached its height. The wards were opened to all. The patients' friends were not admitted till the following day, but there was no lack of people for all that. The night nurses came on at three o'clock and could go just where they pleased and do what they liked. The day nurses took it in turns to stay in the ward while the others went the round of the hospital. All the wards were decorated, each arranged differently, and entertainments of various kinds were going on everywhere. There was singing and reciting—there were Punch-and-Judy shows, ventriloquists, and all sorts of different things. But, most interesting to patients and staff alike, the doctors had dressed up as niggers, singing all manner of ludicrous songs, while there were others like clowns, all in white, who called themselves "The Snowflakes." They dragged their pianolas about from ward to ward, singing and reciting and dancing until they must have been giddy. It was quite exciting trying to make out who they were, beneath the powder and paint and black, for they had disguised themselves most successfully, especially those not blessed with any very prominent features.

### Chelsea Hospital for Women.

On Saturday last Chelsea Hospital for Women was quite *en fête*. The entertainments included a conjuring entertainment and a Christmas-tree. The decorations had been kept up till after this event, and the corridors and wards looked very festive with their rows of fairy lamps, coloured shades, and festoons of evergreens. In the topmost ward all the most convalescent patients were assembled, and here Mr. Charles Bertram gave a display of conjuring which was keenly enjoyed by the patients and visitors. At one stage the ward resembled Tom Tiddler's ground, for Mr. Bertram discovered florins in the most unexpected places, on a nurse's cap, in the air, and on the pillows of the patients. The conjuring over, the patients and visitors adjourned to a ward hard by with a Christmas-tree shining resplendently, at its foot being many neat packages, which, when distributed among the patients, proved to be useful presents provided by kind friends and sympathisers.

Mount Vernon Hospital.

The double doors of Mount Vernon Hospital swung open on Christmas night as wide as ever to admit the visitor, giving entrance without any protecting screen to the dining hall, gay with holly and paper festoons. To the unhardened person the atmosphere seemed hardly in keeping with the celebration in progress. But the patients clearly thought differently. Everyone looked bright and happy—even those who occupied the long open-air balconies that run the full length of the main building, and whose great windows, out of consideration for the visitors, were partially closed. Some thirty of the worst patients were unable to leave their beds in order to join in the general festivities on Christmas Day. But the Christmas dinner party mustered 90 strong in the big hall, and enjoyed two excellent entertainments during the day. Credit is due to the matron (who was appointed only a few months ago), her staff of sixteen nurses, and the assistant secretary for the skill and energy which were expended on the decorations of the hospital and the amusement of the patients.

West Ham Hospital.

The festivities of the season excited the usual enthusiasm at the West Ham Hospital. The decorationswere very simple and pleasing, consisting chiefly of flowers and fairy lights. The approach of Christmas was indicated on the Sunday by the presence of St. John's Choir, who sang carols very sweetly in the wards. On Christmas Eve an "At Home" was held, at which there was a large attendance of visitors to witness the distribution of gifts from the Christmas-tree to the in-patients. The great event of Christmas Day was the dinner, at which the doctors carved, and in the evening a "Magazine Tea" was held for the patients, at which the nurses appeared in costumes representing well-known periodicals.

St. Marylebone Infirmary.

During Christmastide all the wards at St. Marylebone Infirmary were tastefully decorated with evergreens and brightly coloured shades, while various texts and mottoes also adorned the walls. One corridor, by the aid of beautiful flowers and greenery, was charmingly transformed into a representation of Spring. There was competition between the various wards as to which should look the prettiest and possess most novel features. A much-admired group consisted of three Japanese figures snugly ensconced in a geisha tent; this had been constructed by the patients and represented much clever contrivance. A triumph of ingenuity was a motor-car made out of two wheeled chairs, an operating table, and a cover, which was a perfect imitation and had the figure of a man inside. But the palm was readily yielded by all to a large model of a church made by a patient who had been an inmate of the Infirmary for seventeen years, who was almost crippled with rheumatism and could hardly use his hands. In spite of his disabilities, with the help of another patient he had manufactured a most excellent model of a church standing 4 ft. 10 in. high and about 4½ ft. in length. It was largely composed of canvas and pasteboard, possessed a tower with a small spire and a weathercock on top of all, a porch and a clock, and the windows filled with celluloid coloured to represent stained glass gave the whole building when lighted up from within a thoroughly realistic air. Carols sung by the nurses in the female wards were greatly appreciated.

General Lying-in Hospital.

The patients at the General Lying-in Hospital, York Road, Lambeth, awoke on Christmas morning to find a parcel containing a suitable garment and a warm shawl for each baby, with a lucky threepennybit. These were looked at and admired until dinner-time drew near, when chicken and plum-pudding were served with seasonable decorations. A gramaphone lent by a friend was taken into each ward in turn, and played to the delight of the mothers. At six o'clock the lights were lowered and the nurses entered in procession, carrying Chinese lanterns and singing carols. They were followed by Father Christmas, who invited everyone to dip for gifts in his bran tub. By this time the patients were glad to be comfortably settled for the night, and the nurses were free for their evening's pleasure. Dinner was served at seven o'clock, the matron and resident house doctor presiding. An empty ward with a delightfully polished floor was utilised for the evening's entertainment.

### Christmas in Back Streets.

THE CHURCH ARMY AND THE CHILDREN.

Stevenson, in his Christmas sermon, briefly summed up the whole duty of man: "One person I have to make good—myself. But my duty to my neighbour is much more nearly expressed by saying that I have to make him happy —if I may." The Church Army has learnt that.

At Cumberland Mews, a portion of the headquarters of the Church Army, some 150 children between the ages of four and fourteen, were entertained on Saturday, January 5. The basement of the building was used for the purpose, and a more charming place can hardly be imagined, so bright, so gay it was. White glazed tiles are a very good background for holly-sprays and holly-berries, and these, with strings of flags, beneath which were rows of happy faces, made Christmas in the Edgware Road a charming festival. Overhead the crowd was hurrying, but a few children lingered as the laughter and clatter of those below reached them through the open skylight.

There was tea, first of all—such a tea, too! But in spite of every effort the great pile of rock-cakes seemed hardly smaller at the end. Later on the tables were cleared, and games began. These were not difficult to start, partly, of course, owing to the tact of the ladies in charge, and partly because many of the children were friends, for those who did not live in the same buildings had perhaps met at the Church Army's Dispensary. All the guests of Saturday evening had been attended there. The games had not been long in progress—that is to say, the children had not had time to get tired of them—when the ringing of a bell admonished us of the chief feature of the evening. Is there any child who does not like a magic-lantern? A perfect storm of clapping greeted every picture that was thrown on the screen. Dogs, cats, horses which stooped to drink, and sailors who had skipping matches in turn delighted the audience; but these, wonderful though they were, formed but the prelude of the illustrated story which followed. Each event in the story was illuminated by slides, and a running comment was given by someone who stood near the magic-lantern. The best thing about the story was its pathos : children always love sad stories, as those well know who have to invent them. Besides its sadness, however, there was a moral also, which was carefully pointed out to everyone.

This was not all. A Christmas-tree sparkling with presents, soon to be robbed of its strange fruit by willing hands, gave the last pang of satisfaction to the guests. Remember, this was after we had been enjoying ourselves for about three hours! The success of the afternoon was assured. That charity begins at home and should not be allowed to stay there perhaps best describes the way in which the Church Army went to work.

### The Royal National Pension Fund.

### A NEW BADGE.

HER MAJESTY QUEEN ALEXANDRA, as President of this Fund, has approved the issue of a safety-pin brooch, being a reproduction in gold or gilt and red enamel of the badge on the armlet. The price of these brooches is 1s. 7d. and 1s. 1d. respectively, postage included, and they may be obtained from the Secretary at the office. The brooch is to be worn habitually by policyholders of the Pension Fund, who are to reserve the armlet for use upon ceremonial and public occasions only.

### Everybody's Opinion.

### NURSES AND EVENING DRESS.

AN ENGLISH NURSE, signing herself "An Old-fashioned Anglo-Indian," writes to us from India : I have often seen discussions in your valuable paper on the advisability of nurses wearing outdoor uniform, but I have not yet seen a discussion on the question of wearing evening dress in the institute during the few days spent there between cases. I am attached to a private nursing staff in India where it is rapidly becoming the custom for the English nurses on the staff to wear evening dress; and as two or three of us who are Anglo-Indians have not yet adopted the custom, we are regarded as being old-fashioned a[illegible] lacking in modern style. I consider the custom to be [illegible]ravagant and utterly unsuited to occasions where no more than two or three nurses dine together, hence I do not conform to it. My salary is £60 per annum, out of which I must provide all necessary clothing suitable for this climate, supplement my uniform, take annually one month's holiday—which is an absolute necessity here—reserve a little for charity and save a portion for old age. Now in order to accomplish this I find there is nothing left for smart evening dress to be worn regularly in the institute; yet those of us who find ourselves unable to conform to the custom are looked at askance by the more fortunate ones who are ahead of us in these matters. Will some kind practical English nurse help me to portion out my salary so as to include smart evening dress and yet fulfil all the necessary objects stated above? I may add that late dinner in India is a necessity. I shall be deeply grateful to any matron of an institute who will furnish me with advice on the above question and state whether or not evening dress is customary in English nurses' institutes. I have not written merely to cause an amusing correspondence, but to learn from others, although personally I consider the custom absurd.

[We have referred this letter to the ablest nursing authority on the subject we know, who regards the question raised as absurd. She states that no nurses she knows of, while in at her own institution, at home or abroad, are required to wear evening dress. A private nurse usually carries with her ordinary dress, as well as semi-evening dress, for use in cases of mental nursing and also for staying in hotels, where uniform is objected to. Our authority states :—"Our nurses wear evening dress when they are going to the best seats at a theatre or to evening parties, but one would not feel disposed (if asked) to sanction the use of it in the homes, as it would be out of place and far too expensive an arrangement to manage on £60 a year, or even a much larger income."—ED. THE HOSPITAL.]

### PATIENTS' CLOTHING IN HOSPITALS.

"E. W." writes : Will you kindly give me a little space in your valuable paper to reply to "Matron" about storing patients' clothing in hospital? I think that she has used strong language when she implies that I am lacking in ordinary common sense just because I ask if something could not be done for those living long distances from London. Also that she has quite mistaken my remarks about the happy conditions of the patients. I am far too loyal to hospital authorities to wish to insinuate that rules are obnoxious. But let "Matron" give up a whole day of her time to come with a patient who is not able to come alone, and her mother not well enough to come with her, in a pouring wet day, and carry the patient a considerable distance to save expense for the poor mother, and then find the things (by which I mean the whole of the patient's body-clothing and wraps) given her to take away; she might think differently. I understand 11d. is the limit for parcel post. I have far too much sense to expect either infectious or dirty clothing to be retained. If St. Bartholomew's Hospital can manage to keep patients' clothing, why should not others? If nurses would fold skirts up carefully I do not think there would be much need for complaint. I was a patient for five weeks in a large Liverpool hospital some years ago, and I do not remember any creases in my clothing. People living near London would prefer taking the clothing home, so that storage would only be needed for distant ones. In conclusion, I would like to tell "Matron" that, had it been for my own relatives or for people who could well afford to spend money, I would not have said anything, especially as I experienced nothing but kindness shown to me by those in authority both at the time and since when I have had occasion to visit at the hospital. Someone suggested cycles for the Infectious Hospital nurses at Sheffield for recreation, and yet a district nurse is considered lacking in sense if she wants a little consideration shown her when she is helping very poor people with both time and money.

### CHRISTMAS DISTRIBUTION.

THE MATRON of the Shoreditch Infirmary, 204 Hoxton Street, N., writes : I beg to thank you fo[illegible] [illegible]he parcel of clothing you sent to me at Christmas. I [illegible]an safely say that your gift was much appreciated, and has been put to a good purpose. Many of the little ones have been warmly clothed, and made to feel comfortable during the bitter weather we have had. I often feel that it is hard lines for them to give up the comforts they have in the hospital and go back [illegible] want and wretchedness, as it is in many cases.

### Appointments.

MUNDY ISOLATION HOSPITAL, MERTHYR TYDFIL.—Miss D. Williams has been appointed matron. She was trained at the Royal Infirmary, Bristol, and has since been nurse at Perth Cottage Hospital, Rhondda Fever Hospital, and Newport (Monmouthshire) Isolation Hospital.

ROYAL PORTSMOUTH HOSPITAL.—Miss E. Shackleford has been appointed matron. She has previously been assistant matron in the same institution for three months.

### "The Hospital" Convalescent Fund.

THE Hon. Secretary begs to thank Nurse M. Ruel for her kind annual subscription of 2s. 6d., and for her good wishes.

### Workhouse Nursing.

"M. D." writes: May I ask you to afford me space for a few remarks on the subject of Workhouse Nursing from the standpoint of a Poor-law official who is not a nurse? My excuse for troubling you with a communication on so trite a subject must be its importance not only to the nursing profession, but to the sick poor. The British Medical Association has, I believe, collected evidence to bring before the Royal Commission on the Poor-law, and one hopes that sufficient evidence will be produced by those interested in nursing to prove that the existing state of things is unsatisfactory. I refer especially to those workhouses in which the infirmary is not a separate institution, and in which the superintendent nurse is placed in the very difficult position of having to "superintend and control" the other nurses "subject to the directions of the medical officer in all matters of treatment of the sick," and "subject to the direction of the master or matron in all other matters" (see Order of the Local Government Board, August 7, 1897). It is pretty clear that this arrangement is one which can scarcely be expected to work smoothly. A highly-trained professional woman is to superintend her staff of professional women subject to the direction of a non-professional man or woman.

### The Cause of Friction.

I put it bluntly, because I believe firmly that this tactless arrangement is the cause of nine-tenths of that "friction" which Boards of Guardians have so often to deplore. We may safely conclude that the views which different individuals will take as to what are included under "all other matters" will vary considerably; and, human nature being such as it is, we may also conclude that some masters and matrons and some superintendent nurses will take too rigid a view of their rights and duties, and that some will take too exalted a view of their own importance; then, when extremes meet, there will be "friction." If those "other matters" were clearly defined things would work better, but even then the elements of discord would not be excluded. It must be remembered that masters and matrons have very many more opportunities of meeting with and impressing their views upon the Guardians than has the superintendent nurse. It must also be remembered that there is a decided tendency amongst persons of a class from which Guardians are largely recruited to favour the non-professional rather than the professional view, and to ignore, or profess to ignore, special education and training.

### The Anti-Infirmary Party.

Thus in many Boards of Guardians there is a party which may be described as the "Anti-Infirmary" party, composed of men who look back with regret to the old days when the "nurse" was simply one of the master's subordinate officers, employed under exactly similar conditions to the laundress and the task-mistress, both of whom are useful and estimable officers, but not members of a profession with serious responsibilities. Now, I maintain that when certain Boards of Guardians are so wanting in common sense as to pay a high salary to an experienced superintendent and then to place her in an altogether anomalous position, they simply court difficulties. They invest her with the title, but do not give her a firm position, or treat her with the consideration due to the head of a department. She is not consulted in any way about the engagement of new nurses; she is not given a free hand as to arrangement of leave of absence of her nurses; she cannot even arrange which rooms they are to occupy. The Infirmary Committee is attended by the workhouse master, whose opinion is frequently asked upon such matters as the suitability of some particular nurse for such and such a post, while the superintendent nurse is sent for when required, and too often treated rather as a witness under examination than as a trusted and valued officer. I have said that the superintendent is not consulted in the matter of the engagement of new nurses; what actually takes place in at any rate some Unions, is that applications are received by the Clerk to the Guardians, submitted to the Infirmary Committee, who, with the assistance of the Clerk and the workhouse master, proceed to make the appointment, and when the new nurse arrives on the scene she is interviewed first by the master or matron, and finally sent on to her superintendent, who has had no opportunity of hearing anything as to her previous experience or training.

### A Wrong Principle.

It is not Boards of Guardians alone who are responsible for this peculiar state of affairs; the Local Government Board appears to establish the principle that the master is the proper person to judge of a nurse's qualifications, because in Article V. of the above quoted Order it is laid down that in case of emergency, etc., the medical officer is to inform the master that a nurse is required, and "it shall be the duty of the master to engage a person to act as nurse." Now my contention is that this is a wrong principle; the best judge of a nurse is someone who has a practical knowledge of nursing requirements, and that person ought to be the superintendent nurse. Moreover, the duties of workhouse masters and matrons in large establishments are multifarious, and quite enough to occupy both of them fully. The master's time would be much better spent in looking more closely after the work of the able-bodied, the care of the aged and imbecile inmates, the stores, the kitchen, the office, and his other duties too numerous to mention, than in exercising a sort of supervision over a staff of nurses who would be much better left entirely in the hands of their superintendent, as they are in general hospitals.

### The Remedy.

Let Boards of Guardians engage the services of a competent and trustworthy lady for the post; let them by all means exercise the greatest care in making the apointment, but when it is made let them treat their superintendent with confidence, and let the give her an absolutely free hand; then, and then only, will she be able to "superintend and control" the nurses under her charge, and an end will be made of all the gossip and airing of petty grievances which goes on when junior nurses of a certain type find that they can interview individual Guardians, masters, matrons, and clerks, and discuss matters which should be easily arranged without influences from outside.

### Novelties for Nurses.

(By our Shopping Correspondent.)

### "MAGIC": A NOVELTY BY A NURSE.

A special application for the relief of neuralgia and headache has just been brought out by a nurse, who has patented her invention. It is a soft white bandage, fitted with elastic fastenings, which can easily be loosened or tightened at will, the inner side being medically treated. Members of the medical profession are said to speak highly of the bandage, and decidedly the aromatic perfume is refreshing and invigorating. The price is 1s., and it is obtainable from Messrs. E. and R. Garrould, Edgware Road, W.

### A Book and its Story.

AN ENGLISH GIRL IN RUSSIA.*

The author of "The Heart of Helen" has taken for the heroine a young English girl, Helen Lumsden—well born, good looking, and rich, with a highly wrought temperament—for whom Russia and its sorrows has always had an irresistible attraction. Her parents neither understand nor share her interest in the subject, being merely kindly ordinary people living on their country estate, whose claims are a sufficient outlet for their energies. But Helen is weary of the country life and longs for a wider horizon. She wants to travel and see the world beyond the limited circle of interests that surround her. Russia is the bourne of her ambition. An opportunity occurs for her to gratify her longings through the introduction of friends to a Professor Kristofski, who requires an English girl of good position to come out to St. Petersburg as companion to his only daughter, Countess Olga. A salary of £500 a year is offered with the appointment. That this sum was not an inducement that weighed with Helen will be understood when we read that "Helen Lumsden was of age and her own mistress. She had a considerable fortune, amounting to nearly a thousand a year, in her own right. She was therefore an independent woman. Her nature was so amiable, and she had been brought up to love her parents so tenderly, that under ordinary circumstances nothing would have induced her to go against their wishes. . . . For so rich a girl, a girl so absolutely independent, so young, so beautiful, to choose to go as companion to the daughter of a learned professor in Petersburg was in itself incomprehensible." Helen has a friend, Kitty Pembridge, who influences her strongly. Being motherless, she takes the head of her father's house, and Professor Kristofski is a friend of theirs. From them Helen hears of his wish to find a companion for his daughter. Kitty Pembridge has inspired Helen with some of the views which she, as a member of a secret society, holds about the condition of State prisoners in Russia. Helen has thought over the things that Kitty has told her until she becomes fired with the same ambition to sacrifice herself, if need be, on their behalf. Professor Kristofski is staying with the Pembridges, and Helen Lumsden is described at this time: "How tall she was! how gracious! how graceful! how well she carried herself, and what a bright laugh was hers!. . . When in repose her face was grave, but at any instant it could shine into a smile, sweet as sunshine itself." Professor Kristofski is charmed with Helen. He at once recognises that she would be a valuable aid to him. He is a distinguished scientist, holding a post under the Russian Government, subtle, acute, and a mesmerist. At their first meeting Helen's impressionable nature is susceptible to the Professor's magnetic influence. "In the most extraordinary way this radiant young creature seemed to be subjected to his will. He talked to her, and her eyes sparkled, her cheeks glowed with colour, her impressionable heart was roused." He goes away, and upon his arrival in Russia he invites Helen to come out to St. Petersburg. The letter of invitation strikes those who read it as being more in the nature of a command than a request. But the prospect it opens to Helen is an enchanting one: "She must spend a winter in the gayest capital in Europe. She must talk French with the *savants* of the Court. She must go here, there, and everywhere. She must help the professor to introduce beautiful young Olga into Russian society. She would be paid, of course, that went without saying. Helen neither refused the pay nor the invitation. She went, and during her absence she wrote once or twice to Kitty Pembridge and once or twice to her parents. Her letters were full of enthusiasm and rapture." At the end of a year Helen returns home, apparently a nervous wreck: "Changed indeed was the brilliant girl. Pallid was the blooming cheek and the full dark eyes." Then there comes a sudden attack of paralysis, to the sorrow and dismay of her family. But, in spite of apparent helplessness and loss of health and spirits, one absorbing desire possesses the heart of Helen—to return to Russia! When things have reached this crisis Maurice Delafield, who has taken his final M.B. at Cambridge and is starting in practice, comes to stay with the Lumsdens. He has not seen his cousin Helen for two years, and then she was a radiant vision of beauty and health. He finds it impossible to believe that she can be the victim of paralysis. He is deeply in love with her. They had been boy and girl comrades, and the hope of marrying Helen when he was fully qualified had been one that was ever present in his mind. The hypnotic influence under which Helen is suffering very soon makes itself apparent to him. He is looking out of his bedroom window late one night, oppressed by the atmosphere of mystery surrounding Helen. Even the maid, half nurse, also, under whose care she had returned to England was a taciturn mysterious being, and yet "What possible mystery, what possible evil could touch a happy English girl in an English home? . . . The next moment . . . gliding across the grass, moving with perfect ease, as a girl will walk in absolute health, he saw his cousin Helen! With grace and precision she went slowly forward. . . . She walked rapidly, as one in sore need of exercise." By this astounding apparition Delafield is prepared gradually for more exciting experiences. Helen leads him in her infatuation for the cause of Russia nearly to sacrifice his life and her own. He follows her to Russia, where she returns, disguised, with her maid. Countess Olga Kristofski, the professor's daughter, is a remarkable character. She completely dominates her father and the members of his society, the order of the Black Ribbon. Kitty Pembridge realises her personal power on meeting her again in St. Petersburg. "It was quite the coldest, proudest, most inscrutable countenance her eyes had ever looked on. It gave to each fresh person who saw it a strange sense of repugnance, but at the same time it fascinated. . . . At the first glance no one could have accused this remarkable woman of beauty. Her face was deadly white and very heavy in mould." From the professor's study some iron steps lead down to the Neva. The entrance to them is concealed by a panel, which, by touching a spring, discloses the staircase. Through scenes weird and exciting, in which, in a secret underground chamber, the professor and his followers hold their meetings, and to others where, in the brilliant ball-rooms of this city of contrasts, they mingle in the highest society, the reader is borne along till, after all hope seems at an end for the two, by the action of the maid, who opportunely administers sleeping potions to those most actively interested in Maurice and Helen's detention, they make their escape. In spite of the author's desire to make the character of Helen Lumsden that of an ideally self-sacrificing woman, she fails to be convincing. But the story is readable, if only on account of the Russian scenes.

* "The Heart of Helen." By L. T. Meade. (John Long, 6s.)

### Notes and Queries.

**REGULATIONS.**

**The Editor is always willing to answer in this column, without any fee, all reasonable questions, as soon as possible.**

**But the following rules must be carefully observed.**

**1. Every communication must be accompanied by the name and address of the writer.**

**2. The question must always bear upon nursing, directly or indirectly.**

**If an answer is required by letter a fee of half-a-crown must be enclosed with the note containing the inquiry.**

*Nursing in India.*

(165) Can you give me the address of the Up-Country Nursing Association?—*Boscombe.*

This Association no longer exists, but has been merged in Lady Minto's Nursing Association for India. Address the Secretary, Simla.

*Massage.*

(166) Can you tell me of a hospital where I could get a training in massage without paying a premium?—*Basingstoke.*

At the National Hospital for the Paralysed and Epileptic, Queen Square, S.W. Probationers not only pay no premium, but they receive a salary of £12 per annum the first year, and this is increased annually.

*Uniform.*

(167) I wish to train as a nurse. Shall I have to pay for my uniform and laundry?—*Alice.*

Regulations vary at the different hospitals. You had better get "How to Become a Nurse," price 2s. 4d. post free, The Scientific Press, 28 and 29 Southampton Street, Strand, London, W.C.

*Age of Training.*

(168) At what age can I train as a nurse at a London Hospital?—*Bristol.*

None of the large hospitals will receive you before 23.

*Midwifery.*

(169) Can you tell me of a home or institute in London where maternity nurses are employed, and if they can take their own fees?—*Farnham.*

There are several such institutions, but we are not aware that the nurses receive their own fees. Write to the Maternity Nursing Association, 5 Little James Street, Bedford Row, W.C.

*Nursing in India.*

(170) Can you give me the name of the Secretary of Lady Minto's Nursing Association for India, or of anyone I could apply to?—*Reader.*

See answer to *Boscombe.*

*Massage.*

(171) Can you tell me how I can train for massage and electricity in Bristol or Birmingham? Can I train as a resident pupil, and what are the fees, etc.? If you cannot help me as to these two towns, can you recommend an institution in London where nurses are trained at reduced fees?—*Tavistock.*

We cannot help you as to Bristol, but write to Miss Thompson, 36 Strensham Road, Birmingham. Also to the Corporation of Trained Masseuses, 12 Buckingham Street, Strand, London, W.C.

*Nursing of Children.*

(172) Is there any children's home where a well-educated girl of 16 years of age could fill in her time till of age to enter the nursing profession through a regular hospital?—*Steane.*

You had better procure the *Englishwoman's Year Book* (obtainable from The Scientific Press, 28 and 29 Southampton Street, Strand, London, W.C., price 2s. 6d.) and look for Children's Homes. Doubtless by inquiring of the matrons of some of the many homes mentioned you could find what you require.

**Handbooks for Nurses.**

| | Post Free. |
|---|---|
| "A Handbook for Nurses." (Dr. J. K. Watson) ... | 5s. 4d. |
| "Nurses' Pronouncing Dictionary of Medical Terms" | 2s. 0d. |
| "Art of Massage." (Creighton Hale) ... ... ... | 6s. 0d. |
| "Surgical Bandaging and Dressings." (Johnson Smith.) ... ... ... ... ... ... ... | 2s. 0d. |
| "Hints on Tropical Fevers." (Sister Pollard.) ... | 1s. 8d. |

**Of all booksellers or of The Scientific Press, Limited, 28 & 29 Southampton Street, Strand, London, W.C.**

### For Reading to the Sick.

GOD IS LOVE.

MURMUR not at grief,
Find relief
In the love
Which knoweth best!
'Tis God's behest!
Who rules above.

Fear not the morrow
Nor borrow
From its care.
God will provide
Whate'er betide,
He will share.

Therefore come what may,
On each day,
Tranquil rise!
Our God decrees
He ever sees
What is wise.

*Paul Fleming.*

Jesus went straight to His Father with all His troubles. He was not content with any logic of comfort, or any promise of divine good in the final outworking of events. He believed all this, but in His Trial He wanted the blessing of His Father's presence, the warmth of the Father's embrace. Continually we find him fleeing away from the throng, from hatred and persecution, to commune with God. In the hour of His extremest sorrow, while He sought also human sympathy, it was to His Father that He turned for real comfort. "Being in an agony He prayed." Our Master's example should be our guide in every experience of grief or trial. Persuasions, arguments, and promises, however true, precious, and divine they may be, will never bring perfect quiet to a heart in its anguish. We may listen to all that earth's most skilful comforters can tell us even of the consolations of the word of God, but our lonely spirit will be lonely still.

There is a blessing in true human sympathy. God sends our friends to us to bring us little measures of His own love—little cupfuls of His grace. But He Himself is the only true comforter. His love alone is great enough to fill our heart, and His hand alone has skill to bind up our wounds.—*Dr. J. R. Miller.*

Blessed, thrice blessed, if by so giving up ourselves to the guidance of God's Word and Spirit, we become the friends and children of God. For then, when the storms of life burst upon us, we shall have a refuge, a shelter, and a home: we shall have a Friend to Whom we can go, with the blessed certainty of having every trouble hushed, and every tear dried. . . . Thou canst never forget Thine own; Thou canst never leave them alone. Thou wilt guide them with Thy counsel: and after that, receive them into glory. Thou wilt lead them by the green pastures, and refresh their weary souls with the waters of comfort. In life Thou wilt be their friend, in death their hope, and their portion for ever.—*Hugh James Rose.*

